# Sources of Inaccuracy in Photoplethysmography for Continuous Cardiovascular Monitoring

**DOI:** 10.3390/bios11040126

**Published:** 2021-04-16

**Authors:** Jesse Fine, Kimberly L. Branan, Andres J. Rodriguez, Tananant Boonya-ananta, Ajmal, Jessica C. Ramella-Roman, Michael J. McShane, Gerard L. Coté

**Affiliations:** 1 Department of Biomedical Engineering, Texas A&M University, College Station, TX 77843, USA; jfine@tamu.edu (J.F.); klb4333@tamu.edu (K.L.B.); 2 Department of Biomedical Engineering, Florida International University, Miami, FL 33174, USA; arodr829@fiu.edu (A.J.R.); tboon007@fiu.edu (T.B.-a.); aajma003@fiu.edu (A.); jramella@fiu.edu (J.C.R.-R.); 3 Herbert Wertheim College of Medicine, Florida International University, Miami, FL 33199, USA; 4 Department of Materials Science and Engineering, Texas A&M University, College Station, TX 77843, USA; 5 Center for Remote Health Technologies and Systems, Texas A&M Engineering Experimentation Station, Texas A&M University, College Station, TX 77843, USA

**Keywords:** photoplethysmography, cardiovascular disease, remote health

## Abstract

Photoplethysmography (PPG) is a low-cost, noninvasive optical technique that uses change in light transmission with changes in blood volume within tissue to provide information for cardiovascular health and fitness. As remote health and wearable medical devices become more prevalent, PPG devices are being developed as part of wearable systems to monitor parameters such as heart rate (HR) that do not require complex analysis of the PPG waveform. However, complex analyses of the PPG waveform yield valuable clinical information, such as: blood pressure, respiratory information, sympathetic nervous system activity, and heart rate variability. Systems aiming to derive such complex parameters do not always account for realistic sources of noise, as testing is performed within controlled parameter spaces. A wearable monitoring tool to be used beyond fitness and heart rate must account for noise sources originating from individual patient variations (e.g., skin tone, obesity, age, and gender), physiology (e.g., respiration, venous pulsation, body site of measurement, and body temperature), and external perturbations of the device itself (e.g., motion artifact, ambient light, and applied pressure to the skin). Here, we present a comprehensive review of the literature that aims to summarize these noise sources for future PPG device development for use in health monitoring.

## 1. Introduction

**Figure 1:**
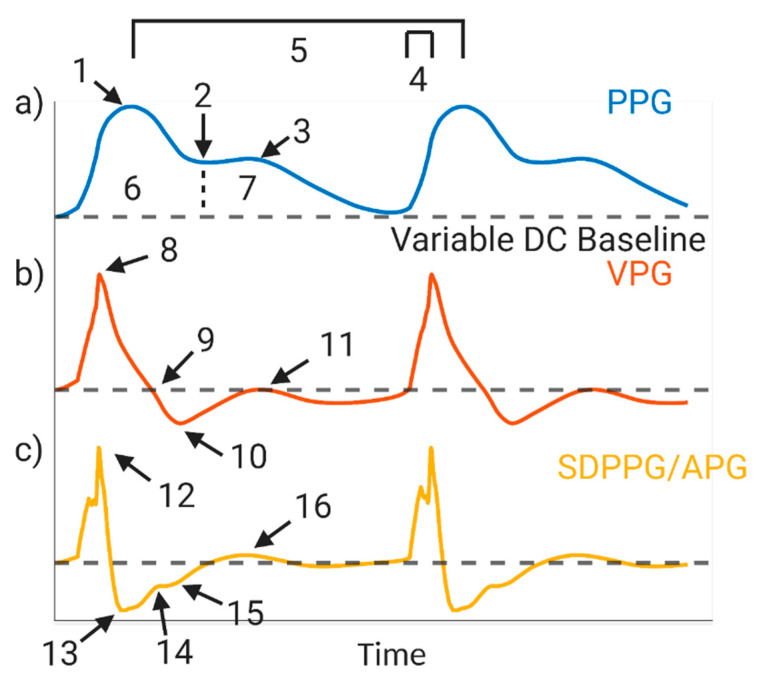
(**a**) Photoplethysmography (PPG) waveform: 1. systolic peak; 2. dicrotic notch; 3. diastolic peak; 4. slope transit time; 5. heart rate; 6. area under systolic waveform; 7. area under diastolic waveform; (**b**) first derivative, velocity plethysmograph (VPG): 8. max slope in systole; 9. end of systolic peak; 10. Start of dicrotic notch; 11. max slope in diastole; (**c**) second derivative, second derivative photoplethysmograph or acceleration plethysmograph (SDPPG/APG): 12. a-point; 13. b-point; 14. c-point; 15. d-point; 16. e-point. Created with BioRender.com.

Remote and continuous/intermittent monitoring (RCIM) has proven to be a promising route to deliver preventative care by reducing both the death rate and burdens placed on the healthcare system [1,2,3]. One emerging RCIM technique frequently being used to monitor wellness is photoplethysmography (PPG). PPG works by illuminating the skin (commonly the finger, wrist, forearm, or ear) with light and collecting the transmitted or reflected light with a nearby detector. The collected light varies in intensity and has a pulsatile component, often called the AC component, and a quasi-DC component. The variation in the quasi-DC component is due to many factors: the optical properties of the tissue, average blood volume, respiration, vasomotor activity, vasoconstrictor waves, Traube Hering Meyer waves, and thermoregulation [4,5,6,7,8,9,10,11,12,13,14]. The common pulsatile (“AC”), change in the PPG is usually the variation associated with arterial blood volume. As the systolic and diastolic pulse travel through an artery or arteriole, the properties of the pulse itself and the compliance of the vessel lead to a change in vessel diameter and consequently a change in blood volume. This correlates with a change in light detected by a photodiode after illumination and hence a change in the voltage or current generated by the photodetector. Changes in erythrocyte orientation can also lead to changes in optical transmittance, further modifying light detected by a photodiode as a function of blood volume [15]. Over an entire cardiac cycle, if the quasi-DC baseline light signal from the other tissue parameters is removed, this leads to the AC PPG waveform, which is attributed primarily to the cardiac pulse. This pulse is often inverted and displayed as seen in Figure 1a. In addition to the possibility of gathering clinical information from the PPG waveform itself, some have used its derivatives to gather information including the first derivative known as the velocity plethysmograph (VPG, Figure 1b) and the second derivative known as the second derivative photoplethysmograph or acceleration plethysmograph (SDPPG or APG, Figure 1c) [16].

Beyond fitness and heart rate monitoring, the primary medical use of the PPG has been focused on obtaining information about the cardiovascular system towards cardiovascular disease (CVD) diagnosis and treatment [17,18]. CVD is a class of chronic conditions and is a general term for those diseases that affect the heart or blood vessels, and include (but are not limited to): coronary artery disease, cardiomyopathy, heart failure, arrhythmia, myocardial infarction, and peripheral artery disease [19]. CVD is often associated with a build-up of fatty deposits inside the arteries (atherosclerosis) and an increased risk of blood clots. It is the number one cause of death globally, contributing to more than 17 million deaths [20]. Cardiovascular disease is currently diagnosed or monitored through noninvasive means using a variety of approaches depending on the specific manifestation. These include: PPG, pulse oximeter, blood pressure cuff, Holter monitor electrocardiagram (ECG), ECG during a stress test, computerized tomography (CT) scans, ultrasound imaging, and magnetic resonance imaging (MRI) [21]. These approaches are often used in combination with monitoring blood biomarkers [22,23]. PPG systems developed for remote and wearable use are typically for general wellness and fitness. This precludes it from being prescribed for medical use at home. The blood pressure cuff, ECG patch, and Holter monitor are also not often used for long-term remote monitoring [24]. Additionally, these tests are rarely administered preventatively, despite research which concludes that preventative testing could reduce deaths by up to 25% [1]. PPG can fill this gap if a sufficiently accurate and precise device is developed.

As depicted in Figure 1, a tremendous amount of information can be extracted from the PPG and its derivative waveforms. Every feature labeled in Figure 1 has been proposed for use to assess cardiovascular health [22,25,26,27]. Specifically, the systolic peak can be used for heart rate, the dicrotic notch and the area of the curve before and after the notch are used for stroke volume, slope transit time can be used for hypertension, the first derivative parameters are largely used to assess blood velocity, and the five points in the second derivative are used ratiometrically to assess vascular health and risk for cardiovascular disease [22,28,29,30]. Additionally, some parameters in the literature such as pulse transit time (PTT), which is used to determine pulse wave velocity (PWV) and estimate blood pressure without a cuff, requires extraction of the time delay from two PPG waveforms or from an ECG and PPG waveform [31,32]. Overall, the literature has demonstrated the potential diagnostic and prognostic strength for the PPG; however, the PPG features can only be utilized if the waveform is of a high quality with high signal-to-noise ratio (SNR).

PPG-based RCIM devices that are U.S. Food and Drug Administration (FDA) cleared or approved and can accurately and consistently record clinical parameters in a sufficiently diverse population for true health monitoring are scarce. Table 1 summarizes the existing PPG-based RCIM devices and their FDA status. All six identified devices with FDA status appear to be able to provide patients and providers with data for oxygen saturation (SpO2), respiration rate, and pulse rate. The oldest device in Table 1 is Equivital™’s EQO2 Lifemonitor, a device worn in a chest harness that also uses ECG. In some cases, FDA approval is only for when the wearable is used within a software suite or healthcare framework. This is the case with the Samsung Gear S2, which is FDA cleared to monitor heart rate toward detecting atrial fibrillation when done with the LIVMOR Halo™ Detection System. The indications for use in these devices are very significant advancements in remote monitoring, but still lag in the potential prognostic capabilities of the PPG. Numerous non-FDA cleared/approved fitness devices exist and can estimate heart rate by quantifying the number of systolic peaks in a period of time, but a single parameter limits the amount of extractable information. Additionally, there are many reports of inaccuracy in these devices [33]. The most popular of these devices are listed in Table 1.

The difficulty in determining features in PPG devices lies in the numerous sources of noise that can impede the output of the PPG. These sources of error pertain to variation within and across individuals (e.g., skin tone, obesity, age, and gender), physiology (e.g., respiration, venous pulsation, body site of measurement, and body temperature), and external perturbations of the device itself (e.g., motion artifact, ambient light, and applied pressure to the skin). In addition, the hardware and software within the device itself can contribute to the noise. These many sources of noise create limitations in the application of PPG to derive advanced physiological parameters. To the authors’ knowledge, there is no work that comprehensively summarizes the literature surrounding these sources of noise and how they affect the waveform of the PPG and its derivatives. Thus, the factors identified in this report may be useful to guide future PPG system designs for true health monitoring. True health monitoring should consider not only the obvious noise sources for commercial fitness devices such as motion artifacts and ambient light, but some of the sources of variability found in diverse patient populations that are prone to cardiovascular disease. These often-overlooked disparities with diversity (e.g., skin tone and obesity) are now becoming more documented in the literature [34,35,36]. Furthermore, this work could assist in defining the parameters that would be needed for human trials to validate the efficacy of constructed devices across variable populations.

## 2. Individual Variations in the Human Population

This section consists of a discussion surrounding works that have explored normal variation within the human population as a source of error or variance within PPG measurements. The variations within the human population to be discussed are skin tone, obesity, age, and gender. These categories largely exist as a spectrum, such as skin tone or age. As such, the effect these categories have on PPG accuracy can be similarly broad.

### 2.1. Skin Tone

**Figure 2:**
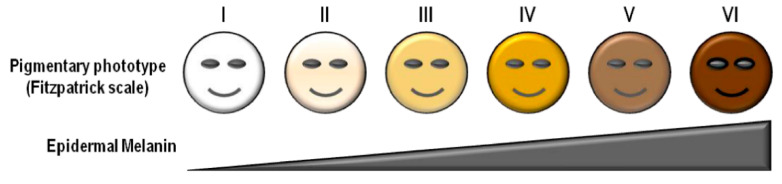
The Fitzpatrick Scale, ranging from I (little epidermal melanin, ~3% volume fraction melanosomes) to VI (significant epidermal melanin, ~43% volume fraction melanosomes) [39].

**Figure 3:**
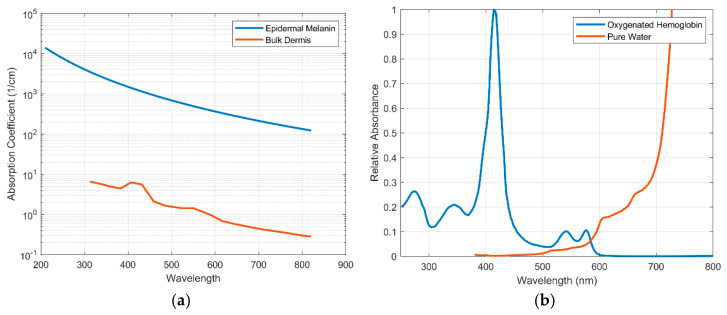
(**a**) Absorption coefficient of epidermal melanin vs. bulk epidermis. (**b**) Optical absorption of oxygenated hemoglobin and water [47,48,49,50].

The most common way to characterize skin tone is via the Fitzpatrick Scale [37]. Shown in Figure 2, the Fitzpatrick Scale ranges from 1 to 6, where 1 is near-albino and 6 is highly pigmented skin. An individual’s skin tone, and thus Fitzpatrick category, is correlated to the amount of eumelanin in their epidermis [38]. While this scale was devised to discuss skin UV-sensitivity, it is often used within the biophotonics community due to the effect eumelanin has on how light travels through skin. This is due to the high absorbance of eumelanin with a peak in the ultraviolet wavelength (220 nm) and a steady decay through the visible wavelength region. Figure 3a illustrates not only this decay across the visible range but also the high, two to three orders of magnitude offset in absorption of epidermal melanin as it compares to the absorption of bulk dermis, which has no melanin. Since the absorption of epidermal melanin is much higher in the visible region of light and much lower in the near infrared region (NIR), the NIR range of light will travel further through pigmented skin. However, many PPG devices use green light (~550 nm). The decision to use green light in most PPG systems is primarily driven by the relatively high absorption spectrum of hemoglobin in this range (Figure 3b), which is the main absorber in blood and thus can potentially give a strong pulsatile signal with changes in blood volume. For those with a lighter skin tone, this enables a higher signal-to-noise ratio for determining heart rate: the primary parameter derived by PPG. However, the wavelength range needs to be optimized for both skin tone and blood absorption, particularly as more advanced parameters are derived from PPG signals, as the absorption of green light by melanin in individuals with a darker skin tone limits the light penetration to the subcutaneous tissue where the blood is located.

Preejith et al. confirmed that skin tone matters when using green light only. The analysis of their PPG biosensor (single source 535 nm LED) showed a significant direct correlation SNR of the heart rate with skin tone, indicating that a darker skin tone yields higher error in the measurement [40]. Specifically, Preejith et al. developed a dorsal wrist-based heart rate monitor that utilizes 535 nm light and validated its performance against 256 subjects (54 with “fair” skin, 181 with “moderate” skin, and 21 with “dark” skin). The device features a single green LED and two photodetectors on opposing sides. Ground truth was obtained using a Masimo Radical-7 which is a commercial handheld fingertip pulse oximeter that uses seven wavelengths of light across the visible/NIR range to determine cardiovascular parameters. The Masimo Radical-7 was placed on the index finger of the same hand where their device was worn. Their results indicate a greater than 10 times increase in absolute error with the darker skin tone, calculated by taking the absolute value of the difference between their sensor and the Masimo Radical-7. There was an error of 1.04 beats per minute (BPM) for “fair” skinned individuals and an error of 10.90 BPM for “dark” skinned participants, citing the lack of usability of their device for dark skinned individuals. Hermand et al. determined the same trends as Preejish et al. while using the PPG heart rate monitor Polar OH1. They analyzed its performance across 70 subjects ranging from Fitzpatrick 1 to Fitzpatrick 6 during various levels of exercise and motion. Hermand et al. had participants run, bike, and walk while wearing the Polar OH1 on their upper arm and the Polar H7 chest strap (ECG based device) paired with the Polar M400 watch as the ground truth. The Polar OH1 consists of 6 green LEDs forming a circle around a single photodiode. In determining heart rate, it was found that bias (defined as the mean difference between the OH1 and ground truth) increased with darker skin (*p* < 0.001), and heart rate accuracy was positively correlated to skin tone (*p* < 0.05). However, the authors also mentioned that the lack of environmental control leads to increased humidity and possibly increased vasodilation [41]. In addition to these examples, numerous other studies that stratify results against Fitzpatrick classification identify the same trend; errors in determining heartrate from wearable PPG devices that use primarily green light as their source is increased in individuals with dark skin tones due to the high absorption caused by increased amounts of epidermal melanin [42,43,44,45]. Lastly, Bent et al. published an experimental analysis of error observed in optical heart rate sensors manufactured by Apple (Apple Watch 4), Fitbit (Fitbit Charge 2), Garmin (Garmin Vivosmart 3), Xiaomi (Xiaomi Miband 3), Empatica (Empatica E4), and Biovotion (Biovation Everion) as a function of skin tone [46]. By collecting data from 56 patients (34 female, 22 males, 18–54 years old, and at least 8 participants in each Fitzpatrick classification) when they are at rest and exercising (elevating heart rate to 50% of maximum via a treadmill), it was found that there was no statistically significant relationship between measured heart rate from the wearable and an ECG (Bittium Faros 180) reference. Interestingly, this conclusion contrasts with the previous discussion. It is possible that the lack of increase in error observed is due to the already large error present in the results reported—the mean absolute error is approximately 9 BPM across all skin tones. The authors do not discuss why the results presented conflict with those previously reported. Additionally, the wearable devices used in this study utilized red and near infrared light, which can more easily penetrate the epidermis.

The lower absorption of melanin using light sources at higher wavelengths can improve the signal for individuals with a high Fitzpatrick classification. Mohapatra et al. demonstrates that this is the case with a multiwavelength PPG device placed on the central dorsal wrist. The device comprised 2590 nm (yellow/orange) LEDs and a single 520 nm LED symmetrically opposite to the 590 nm LED on the vertical axis. There was approximately 0.7 cm center to center source/detector separation for each LED. With 20 subjects ranging from Fitzpatrick II to Fitzpatrick IV, the perfusion index (AC/DC), pulsatile strength, and SNR were all found to increase when data collected with the 590 nm LED were analyzed. For Fitzpatrick IV individuals, the perfusion index increased between 1.2 and 7.1 times with the use of the 590 light, while pulsatile strength increased by 1.1 to 3.1 times and SNR increased by 1.3 to 2.6 times, although no statistical significance analyses were performed [51]. Fallow et al. performed a similar study by using more wavelengths and including exercise [52]. Specifically, Fallow et al. measured reflectance PPG above the radial artery (4 cm from the wrist), that likely included a signal from both the arterioles and possibly the artery depending on the wavelength of light used, in 22 individuals with varying skin tone from Fitzpatrick scales I to V. By obtaining a resting signal then having participants exercise via forearm flexion and extension, researchers were able to determine the SNR of participants with four different wavelengths (blue—470 nm, green—520 nm, red—630 nm, NIR—880 nm) at rest and after exercise. Mean modulation, often termed the perfusion index, defined as the ratio of AC/DC, was significantly lower in Fitzpatrick V individuals than others for all wavelengths (*p* < 0.001). In the resting condition, green light had larger mean modulation (*p* < 0.001) than other wavelengths, and after exercise, blue and green had greater SNR ratios than red or infrared (*p* < 0.001). These results indicate that overall, the mean modulation goes down with increasing Fitzpatrick scale, and with exercise one would expect greater blood volume changes causing a higher signal for the more absorbing blue/green wavelengths, at least for the source to detector distances used in the study. Further research should be conducted that analyzes the fidelity of the PPG waveform under these conditions, which would allow interpretation of which wavelengths can be used to derive more complicated parameters from PPG [52]. Furthermore, since no details are given on the source/detector alignment of these devices, different source/detector alignments should be assessed as that can dramatically affect the performance of PPG devices depending largely on the wavelength of light due to light–tissue interactions. This is seen in Mendelson et al., who analyzed the effect of source–detector separation on pulse oximetry with red (660 nm) and infrared (950 nm) light [53]. By collecting the SpO2 of seven Caucasian, lower Fitzpatrick scale, individuals via a Hewlett-Packard HP47201A transmittance eight-wavelength ear oximeter and reflectance (red and NIR) oximeter on the left thigh, the effect of source/detector separation on the reflected light was determined [53]. It was found that relative amplitude of the AC component to the entire signal (AC + DC) increased as LED/photodiode spacing increased from 4 to 11 mm, although no statistical analyses were presented. Subsequent studies should analyze the relative performance of various wavelengths on different skin types, but ensure that data at each wavelength are collected at an optimized source/detector separation. This could be facilitated first in silico via modeling using Monte Carlo Simulation [54,55]. Furthermore, one component of optimizing source/detector separation is the depth of the target vessel and intervening pulsating arterioles, which may vary across individuals and within individuals with body location and due to factors such as level of obesity [56].

### 2.2. Obesity

Obesity, determined by having a body mass index greater than 30, affects 40% of the United States and leads to cardiovascular disease, hypertension, and type 2 diabetes [57]. It is caused by a combination of physical, behavioral, environmental, and genetic factors that lead to the accumulation of body fat [48,58]. In particular, obesity can lead to changes in skin thickness, blood flow, and oxygen saturation. This affects the optical properties of skin in addition to the distance light has to travel to reach a target vessel or vessels [48,59,60]. The variation of BMI across individuals is thus a potential source of variation for PPG measurements. While, to the authors’ knowledge, publications that experimentally and explicitly demonstrate the effect of obesity or BMI on the PPG waveform are limited, we will explore works which suggest that there would be a substantial effect [56,59,60,61,62,63,64,65,66,67,68,69,70].

Blood flow regulation and oxygen saturation are both known to deviate with respect to obesity and BMI [71]. Individuals with obesity experience increased cutaneous blood flow to meet the oxygenation needs of tissue. However, the blood flow of adipose tissue generally decreases with obesity both after a meal and during a fasting state [61,72]. Conversely, Chin et al. used laser doppler flowmetry and dynamic capillaroscopy to measure cutaneous blood flow at the nailfold of children of comparable age, sex, and skin temperature, but with different levels of obesity, finding significant increases in baseline cutaneous flow with obesity [61]. Dermal blood cell flow has also been shown to significantly increase in the forearm of overweight individuals (BMI 29.1 ± 2.7 kg/m^2^) compared to non-obese (BMI 20.4 ± 1.9 kg/m^2^). In adults, while some studies show that at rest there is no significant change in dermal capillary density, the majority of findings find that dermal capillary density does negatively correlate on average with increasing BMI [62,63,64,65]. This effect of obesity could lead to a decrease in the dominant “DC” component of a PPG waveform due to the increased blood volume of the obese [61,62,63,64]. The literature has confounding results in capillary recruitment, defined as the percentage increase in capillary density during venous congestion, in the obese, but this could be due to the populations studied. For instance, Czernichow et al. reported capillary recruitment in the skin after adjustment for age, sex, mean arterial pressure and fasting glucose was higher in overweight (defined as BMI 27.9 ± 2.7 kg/m^2^) as compared with lean individuals, and that obese individuals were normotensive, nondiabetic, male and female subjects [63]. However, the findings of De Jongh et al. showed capillary recruitment to be decreasing rather than increasing with obesity (defined as BMI > 30 kg/m^2^), but the study was done only on women with a mean age of 38.9 ± 6.7 years and the obese subjects were both hypertensive and had impaired insulin sensitivity [65]. In this study, it was acknowledged that capillary recruitment was negatively correlated with blood pressure but positively correlated with insulin sensitivity [57]. Although higher capillary density on average appears to negatively correlate to BMI, the subject’s BMI level, gender, age, as well as their metabolic syndrome need to be considered when assessing capillary recruitment. A hypothetical increase in capillary recruitment due to an increase in BMI would similarly decrease PPG signal intensity, as is hypothesized for dermal capillary density. Lastly, oxygen saturation of hemoglobin has consistently been shown to be inversely associated with BMI across various populations [66,67,73]. The increased absorption of deoxygenated hemoglobin compared to that of oxygenated hemoglobin in the far-red region could decrease SNR of PPG measurements that use those wavelengths of light. The cumulative effect of various vascular changes in the obese on the PPG waveform remains to be seen. One such change where the impact on PPG has been observed in silico is that of skin thickness.

Perhaps most detrimental to the PPG waveform is skin thickness, as it is directly correlated to BMI and can dampen PPG signal amplitude [56,70]. For example, the epidermal thickness of the volar forearm has been shown to be higher in overweight normotensive nondiabetic individuals compared to age- and sex-matched healthy controls [72]. Note that skin thickness in the literature has always showed an increase with obesity, but the increase is body site-dependent. To the authors’ best knowledge, literature does not exist that demonstrates this relationship for the finger, a common location for pulse oximeters. Elsewhere, this thickening leads to a dramatic effect on the physiology and structure of the skin, consequently reducing the signal strength and resolution as photons encounter more possibilities for scattering, absorption, and autofluorescence with thicker tissue [74]. For example, the increase in skin thickness affects the vessel depth, and Boonya-ananta et al. used Monte Carlo simulations that predicted a 40% loss of PPG signal amplitude due to this effect in obese individuals, specifically on the wrist as the simulated radial artery increases in depth from 2.5 to 3.5 mm [74]. This loss of PPG signal amplitude is significant as it makes it more difficult to quantify and identify features within a waveform.

One observation regarding the skin of the obese which may serve to increase PPG signal intensity is the increase in trans epidermal water loss (TEWL) as BMI increases [69]. At longer wavelengths, the absorption of water is more dominant than hemoglobin (Figure 3b). Overall, the morbidly obese exhibit a reduction in water, measured as an increase in the TEWL compared to normal BMI subjects [69]. This in turn could increase the signal component in the NIR and IR range due to the reduction in water molecules. Interestingly, however, the TEWL values in the epidermis of the face, forehead and abdomen decrease from normal to overweight but then increase from overweight to obese and morbidly obese [69]. Due to these inter-dependencies, measuring capillary densities, blood flow, and oxygen saturation in the obese using optical signals should be validated using other non-optical modalities before a definite conclusion can be made on how these parameters impact the PPG signal.

While the exact cumulative effect of these BMI-related parameters on PPG remains unknown, Ferdinando et al. were able to identify obesity from PPG waveforms originating from the Liang et al. dataset [75,76]. Using k-nearest neighbor and support vector machines, Ferdinando et al. were able to identify five classes of obesity from PPG waveforms using 17 parameters derived from the decomposition of the PPG waveform into five lognormal functions. While arterial stiffness is mentioned as a potential cause of variations in the PPG waveform, the work does not discuss characteristics of the waveform itself, or how a waveform originating from an obese individual compares to a waveform originating from a non-obese individual.

Overall, the literature has shown that obesity dramatically affects physiological factors associated with PPG signal intensity and quality, including capillary density, capillary recruitment, blood flow, SpO2, TEWL, and skin thickness. These changes are summarized in Table 2. There are also in silico works suggesting that obesity can dampen PPG signal intensity. We believe that the aforementioned literature provides strong evidence that obesity, when assessed in combination with a subject’s metabolic state, body location, gender and age will likely influence PPG systems and can manifest themselves as significant noise sources. The next section will go into more detail about the later, chronological age.

### 2.3. Age

Aging leads to various anatomical and physiological changes that impact the ability to use PPG to assess cardiovascular health. These changes mostly occur in the vascularization. As arteries age, the tunica intima and tunica media layers within the arteries thicken with an increase in the number and density of collagen [78]. The cross-linking of these fibers, along with fractured and fatigue elastin, leads to a loss of compliance and increased artery stiffness [78]. Along with calcification, the result of these age-related changes is an increase in blood pressure, observed in older populations [79]. Additionally, the endothelium of the vessels thickens and develops irregularly shaped cells over time which increases blood flow resistance [78]. PPG principally measures the response/elasticity of arteries to blood flow, and thus will change if the properties governing artery compliance changes. Beyond vascular changes, skin thickness is another parameter that has a relationship with age. Once adolescence is reached, skin is known to thin as age increases [70,80,81,82]. This relationship is maintained in the three primary layers of skin: epidermis, dermis, and hypodermis/subcutis. Thinning represents a decrease in distance for light to travel before it interacts with vessels, possibly affecting PPG [70,80,81,82]. Overall, these changes can manifest themselves in variations observed across PPG waveforms either in the shape or amplitude of the waveform.

There are many components to a PPG signal and its derivatives that literature records having variation as a function of age [83]. There are differences in the timing of events, manifested as changes in parameters such as PTT, and relative amplitude of features such as the dicrotic notch. Many of these result from changes in vessel compliance, as the decrease in distensibility of arteries leads to different changes in blood volume when compared to younger individuals.

**Figure 4:**
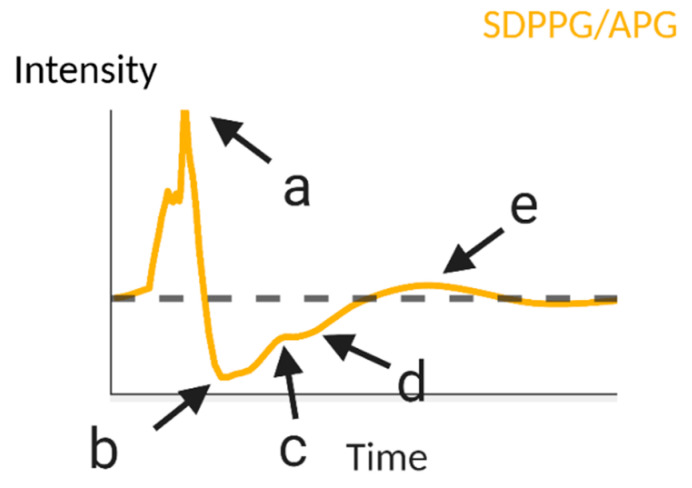
Five primary points of interest in an acceleration plethysmograph (PPG 2nd derivative). Created with BioRender.com.

Using data from 93 individuals of various ages, Ahn et al. extracted features from fingertip-based PPG and the second derivative PPG, acceleration plethysmography (APG, Figure 4), and correlated them to chronological age in order to determine what they defined as the vascular age index [83]. However, this is not to be confused with vascular age, which is a specific term used to guide risk assessment for CVD, and is well known from D’agostino et al.’s work, often referred to as the “Framingham study” [84]. The work of Ahn et al. more closely resembles a chronological age index, as they correlated PPG features to chronological age and not CVD risk [85]. The parameters listed in Table 3 attributed to Ahn et al. yielded a statistically significant, albeit poor, correlation with age [83]. Others have also reported on correlations involving the APG. The ratio *b*/*a* has been found to correlate positively to age, while *c*/*a*, *d*/*a* and *e*/*a* have all been correlated negatively to age [29,59]. Jayasree et al., Dutt and Shruthi, and Yousef et al. similarly report changes in the PPG and APG, such as an increase in the area under the systolic peak with increasing age, a decrease in time between the systolic and diastolic peaks with increasing age, and an increase in crest time as age increases [86,87,88]. While these works relate features within a waveform to age, they do not examine characteristics that are derived from multiple PPG waveforms such as PTT and PWV.

The timing of blood transit through vessels is an important measurement used to determine clinically relevant parameters such as heart rate variability and blood pressure. Caceres and Hasan used the first derivative to create the “waveform transit time” index. This index utilizes an inverse linear relationship to correlate age to the manually estimated difference between the first local minimum (occurring at the systolic peak) and the next local maximum (occurring at the dicrotic notch) of the first derivative. This time difference was termed TT_W_. The index was created by using data from the right index finger of 230 Spanish subjects (134 male) ranging in age from 8 to 89 years old. It was found that TT_W_ decreases with age [89]. The authors state that this is a function of the time delay associated with incident and reflected light in a PPG, and is an easy way to determine PWV, which has been shown to decrease as a function of age. Using the ears, fingers, and toes, Allen and Murray analyzed the changes in PPG signal normalized width and PWV as a function of age. Corroborating with other literature, an increase in pulse wave velocity with age is related to a diminished dicrotic notch. They also note the increase in time of the systolic rising edge (and thus decrease in slope of the systolic rising edge) as a function of age. This is explained by the increase in resistance and decrease in compliance of older arteries [90]. PTT is inversely related to PWV—the same authors note in a different work that as age increases, PTT decreases. By analyzing the change in PTT as a function of age within a population of 134 healthy, Caucasian subjects (median age 43, total range: 13–72), it was found that there is a statistically significant decrease at the toes (r^2^ = 0.48), fingers (r^2^ = 0.26), and ears (r^2^ = 0.15) [91,92]. Most of the listed changes are due to the cardiovascular system, as arteries change how they respond to a cardiac pulse as the vessel ages. However, the integumentary system also responds to aging.

Skin thins as age increases beyond adolescence [70,80,93]. Keratinocytes become shorter, water content decreases, lipid content decreases, and most notably, collagen synthesis and turnover decrease [93]. This change in skin thickness decreases the necessary propagation of light to obtain a PPG signal, which can contribute to an increase in PPG signal strength, along with capillary depletion. Leveque et al. studied the skin thickness and PPG signal of 69 individuals from 8 to 89 years old [94]. A Holtain skin caliper applied to the dorsal forearm found a decrease in skin thickness, and an infrared PPG was used to determine PPG amplitude. The authors attribute the positive, direct relationship between PPG amplitude and age to capillary depletion, due to similar skin thicknesses observed between older participants and children, despite an increased PPG amplitude in the older participants. However, the skin thickness data presented in this study are not consistent with those in other literature, so further work is required to be conclusive. Another study by Hartmann et al. determined that there is no statistically significant change in PPG amplitude as a function of age; however, this work featured 36 individuals ranging in age from only 33 to 58. It is possible that 25 years may not be long enough to discern a significant change [95]. In a study with participants ranging in age from 30 to 60+, Yousef et al. found that there is no significant increase in pulse magnitude measured at the index finger with an increase in age. This work suggests that the decrease in vessel compliance will contribute more to variations in a PPG waveform than other effects of aging [88]. 

Overall, the impact of age-related changes in skin thickness on PPG may be dependent on the body site of measurement, and its significance is yet to be directly studied. However, if this effect is delineated and determined to be significant, it can be used in models that aim to determine blood pressure from PPG.

Artificial intelligence and machine learning have been used to determine blood pressure from PPG and mitigate the effects age have on the estimate. In 2010, Monte-Moreno utilized machine learning to determine blood pressure from a PPG obtained from subjects ranging from 9–80 years old. An algorithm that incorporates age into systolic and diastolic blood pressure determination was created. While this work utilized a population of individuals with good cardiovascular health, it was found to correlate with both systolic and diastolic blood pressure, with an r^2^ of approximately 0.90 [92]. Suzuki and Oguri similarly used artificial intelligence to determine blood pressure from a cuffless monitor and incorporated age into their algorithm [96]. While the wide spectrum of ages can make it a difficult parameter to incorporate into models, gender is binary, making it easier to incorporate.

**Table 3:** Age-induced changes on PPG.

Parameter	Change as Age Increases	Relevant Work	Reference
**Tissue Changes**			
Skin thickness	Decrease	Derraik et al., 2014 Van Mulder et al., 2017 Farage et al., 2013	[70,80,93]
Artery compliance	Decrease	Knight et al., 2017 Allen et al., 2002	[78,90]
Capillary Recruitment	Decrease	Leveque et al., 1984	[94].
**PPG Changes**			
PWV	Increase	Cáceres et al., 2015	[89]
PTT	Decrease	Allen et al., 2002 Monte-Moreno, 2011	[91,92]
Systolic rising edge slope	Decrease	Allen et al., 2003	[90]
Dicrotic notch shape	Decrease	Allen et al., 2003	[90]
Systolic time	Decrease	Ahn et al., 2017	[83]
Diastolic peak amplitude	Decrease	Ahn et al., 2017	[83]
Inflection point area	Decrease	Ahn et al., 2017	[83]
Reflection index	Increase	Ahn et al., 2017	[83]
**First Derivative Changes**			
TT_W_	Decrease	Cáceres et al., 2015	[89]
**Second Derivative Changes**			
Magnitude of *c*	Increase	Ahn et al., 2017	[83]
Magnitude of *d*	Decrease	Ahn et al., 2017	[83]
Slope of line between *b* and *d*	Increase	Ahn et al., 2017	[83]
*b*/*a*	Increase	Ahn et al., 2017	[83]
*c*/*a*	Decrease	Takazawa et al., 1998 Elgendi et al., 2012	[29,59]
*d*/*a*	Decrease	Takazawa et al., 1998 Elgendi et al., 2012	[29,59]
*e*/*a*	Decrease	Takazawa et al., 1998 Elgendi et al., 2012	[29,59]

### 2.4. Gender

The physiological differences between men and women extend into cardiovascular health and are thus noticeable in the PPG waveform [97,98]. Phuong and Maibach noted that gender differentiation in average blood pressure and average heart rate indicate that baseline differences in vascular and cardiovascular parameters must be considered to determine cardiovascular health between males and females, which also extends into analyses of the PPG waveform [98]. These physiological baseline differences can skew PPG signals along with interpretation algorithms for the determination of cardiovascular health per gender. Proctor et al. reported that the average heart rate for males was 70–72 BPM and the average for females was 78–85 BPM in 16 endurance-trained men and 14 endurance-trained women [99]. Males have a 15–30% increase in heart mass compared to females resulting in females’ hearts having to beat faster to maintain the same output [100]. This leads to dramatic changes in PWV and subsequently PTT. Regarding blood pressure, Reckelhoff reported the mean blood pressure was 6–10 mmHg higher in males than in pre-menopausal females. However, post-menopausal females have higher blood pressure than males, which can be attributed to arterial stiffness [101]. Additionally, most vessels have gender-dependent diameters. For example, the radial artery diameter also differs between males and females, with males having a diameter of 2.76 ± 0.009 mm and females a diameter of 2.32 ± 0.07 mm at the same segment [102]. As expected, a larger target vessel will yield a PPG with a greater signal resolution. These significant differences in artery diameter lead to significantly different radial artery flow rates as well, with a flow rate of 21 ± 4 and 10 ± 1 mL/min for males and females, respectively [102]. Skin thickness is another gender-dependent variable known to affect signal strength (as mentioned previously), and is observed to be higher in men than women across all age ranges [97,103]. Shuster et al. measured skin thickness in 90 Caucasian men and 107 women and found that forearm skin thickness is greater in men than women, but no statistics were presented to explore statistical significance [103]. Each of the aforementioned parameters can influence the hardware chosen and utilized when designing a PPG system to ensure a normalization between measurements for males and females, as they directly translate to a difference in PPG signal.

PWV is highly dependent on vascular stiffness and is observable through PPG. Ahimastos et al. studied the changes in arterial stiffness in pre- and post-puberty males and females by measuring arterial compliance and PWV [104]. It was discovered during prepuberty, females have lower arterial compliance and higher central and peripheral PWV yielding stiffer arteries compared to males. Post-puberty, both males and females have an increase in arterial compliance, determining no gender-dependent differences between the measurement. However, for females, PWV stayed roughly the same, while there was an increase in PWV for males. This increase in PWV originates from an increase in arterial stiffness for males corresponding to a higher pulse pressure while the female pulse pressure remained constant throughout pre- and post-puberty. Pulse pressure is dependent on both arterial stiffness and cardiac output. On the other hand, post-menopausal females experience an increase in arterial stiffness and pulse pressure. Dehghanojamahalleh et al. demonstrated variations in PPG morphology attributed to gender differences [105]. Interestingly, direct influence of gender variation was only significantly different at the upper peripheral measurement site such as the hand and fingers as opposed to lower extremities such as the ankle and feet. The study measured the pulse arrival time and PTT, where both measurements showed dependence on gender. Pulse wave propagation delay between the genders indicates baseline differences in arterial stiffness with women displaying lower degrees of pulse arrival latency delay indicating higher vascular stiffness [106,107]. A study on heart rate variability using PPG by Antelmi et al. shows changes between gender across different age ranges [108]. The results presented show men as having greater low-frequency signal components and women as having greater high-frequency components. When comparing the accuracy of commercial devices as done by Shcherbina et al., a significantly higher device measurement error is seen in males than females for all devices [109]. The devices under analysis include the FitBit, Apple Watch, Microsoft Band, Samsung Gear, and Basis Peak watch, and the measurement metrics showing higher error for males include heart rate and maximal oxygen uptake. This provides insight into PPG signal variation due to varying deep internal vasculature leading to peripheral measurement sites.

Accounting for the manifestation of these differences in a PPG is known to be an under-researched area [110,111]. For respiratory measurements, Nilsson et al. have reported that respiratory synchronous variation in the PPG signal is irrespective of gender, and thus no action is required [111]. Nowara et al. report insignificant differences in blood volume pulse SNR in iPPG (imaging PPG) between males and females [112]. More work should be conducted to evaluate accountancy methods for the propagation of gender-induced variation in applications of PPG beyond respiration. Table 4 summarizes the physiological data supporting the presence of differences.

## 3. Physiology

While the previous section discussed variations across individuals, the next section will discuss the effect that physiology can have on the PPG waveform. We will discuss respiration, venous pulsations, body site of measurement, and local body temperature. Underlying physiology can affect the baseline values or periodicity of the PPG waveform or even change the waveform shape entirely [115,116]. Thus, it is important to explore and identify these sources of error so that they do not propagate to cardiovascular parameter values.

### 3.1. Respiration

While the most commonly examined component of the PPG signal is the AC component relevant to pulsatile blood volume, there are various factors which can modulate the baseline of the PPG signal; one such factor is respiration. This is one of the most significant sources of error in heart rate measurements using PPG, even though respiratory rate is the most sensitive vital sign, often used as an indicator of clinical deterioration [117]. PPG optical signal modulation by respiration is most commonly manifested as baseline and amplitude modulation [117]. Physiologically, respiration and cardiac output are inherently linked, as an increase in respiration rate can directly affect the variation of heart rate through the nervous system reduced inhibitory control [118]. Modulation of the baseline due to respiration presents itself as a superposition of the PPG signal corresponding to the cardiac cycle and a lower-frequency sinusoidal waveform [117]. It is reported that the lower-frequency wave manifestation in the total PPG signal can be attributed to the venous vascular system [119]. As respiration occurs, the venous system is more compliant to smaller changes in pressure as opposed to the less distensible arterial system [118]. Shifts in blood volume in the venous system due to changes in respiratory behavior will cause a corresponding change to the baseline amplitude of the PPG signal as blood volume increases and decreases [120]. Changes in thoracic volume and pressure cause an alternating pressure gradient in the venous vascular system [118]. It is also suggested that the coupling of the respiratory system and the autonomic nervous system is a contributor to mechanical effects on the vascular system which is detected in the PPG waveform as a direct result of respiration. Removal of this signal contribution is often done via filtering and post processing.

**Figure 5:**
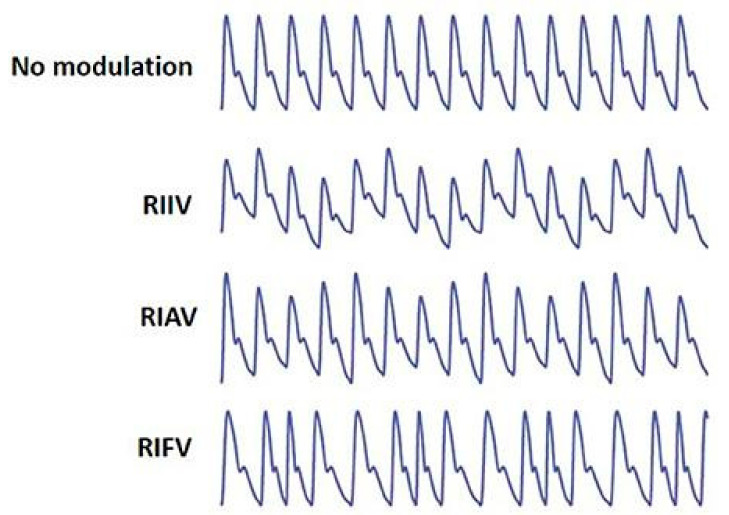
Respiratory-induced intensity variation (RIIV), respiratory-induced amplitude variation (RIAV), and respiratory-induced frequency variation (RIFV) respiratory-influenced variation to PPG signal [115].

There are a wide variety of studies looking to extract respiration rate from obtained PPG signal. However, it is, first, critical to investigate the quantifiable changes and errors to the PPG signal caused by respiration rate [118]. Dehkordi et al. and Addison et al. identified the three significant variations to the PPG signal by respiratory rate after analyzing PPG from 139 healthy adults (67–18 years old) and the Capnobase dataset [115,121]. These variations are respiratory-induced intensity variation (RIIV), respiratory-induced amplitude variation (RIAV), and respiratory induced frequency variation (RIFV), as shown in Figure 5 [115,121]. RIIV modulates the DC baseline of the PPG curve superimposing the wave on top of a low frequency sinusoid. RIAV causes significant changes in each peak amplitude. RIFV induces a phase shift between each cycle by elongating or squeezing each wave. These changes to the PPG signal can occur in any combination.

Li et al. conducted an investigation on the respiratory induced variations to the PPG signal with a total of 28 subjects, 14 male and 14 female, with age ranging from 18 to 45 years [122]. The experiment was conducted with various controlled breathing conditions, comparing period and amplitude of systolic, diastolic, and overall wave cycle. Across the different positions, amplitude correlation coefficient shows distinctly larger difference between diastole and reference versus systole and reference. However, amongst the various conditions, the three factors that present the strongest correlation with respiration rate influence are pulse period frequency variation, diastole amplitude variation, and peak intensity variation [122]. From experimentation results, it is concluded that respiration signal amplitude has the strongest influence on respiratory-induced variation of the PPG signal. Moreover, as the respiratory rate increases, the respiratory signal decreases, indicating that higher rates of respiration leads to lower fluctuations in respiratory influence changes to PPG [122].

Through understanding the main influence of respiratory rate on overall PPG signal, techniques can be developed to target these changes to separate the two signals. As a method of isolation of the PPG signal from noise caused by respiration, some different mathematical methods of signal analysis and conditioning have been proposed. The basic method of frequency filtering has been used to eliminate the frequency components contributed by respiration [118]. Typically, high-pass filters with cut-on values ranging from 0.25 to 0.5 Hz are used to eliminate low frequency noise which is most often representative of respiration rate. Various other studies have been conducted to extract and separate respiration rate from PPG signals using different algorithms or complex neural networks [117,123,124].

Charlton et al. confirmed that the extraction of respiration rate from PPG and ECG measurements is possible by testing 314 different extraction algorithms operating both in the time domain and frequency domain [117]. However, the results indicate that respiration rate extraction is more precise when performed on ECG data as opposed to PPG. It is suggested that this is due to the physiological mechanisms which generate these two signals and their unique interaction with respiratory rate. The mechano-physiology factors that lead to the behavior measured by PPG appears to be more sensitively influenced by respiratory modulation. Interestingly, algorithms operating on the time domain provided more accurate results than frequency domain extraction [117]. It is indicated that time domain algorithms do not require quasi-static respiratory rate, unlike frequency domain algorithms, which may contribute to their superior performance [117]. As mentioned previously respiration is often filtered out and classified as noise. However, in cases where this is desirable information, one looks towards the AC component of the venous network.

### 3.2. Venous Pulsations

**Figure 6:**
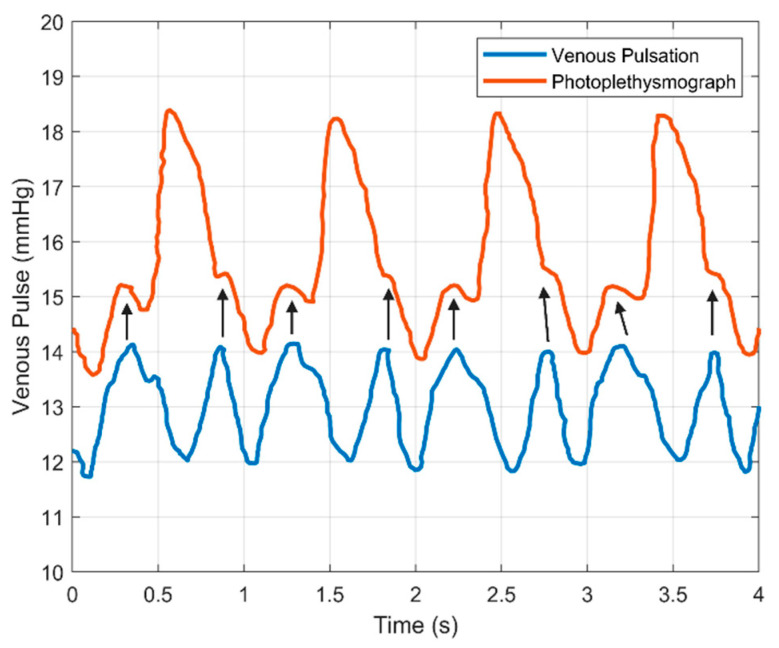
Venous pulse within a PPG. The blue waveform is the venous waveform, and the red waveform shows the PPG waveform. Adapted from Shelley et al. [116]. Created with BioRender.com.

As discussed at length, PPG sensitivity to blood volume changes yields an “AC” signal component that relates to the mechanical properties of a corresponding vessel and even the larger cardiovascular system. The venous system similarly contributes periodicity to the PPG signal, but this is often considered noise. This noise originates from the vascular network of small vessels transporting deoxygenated blood from the capillaries to the heart. However, it is a recognizable waveform (Figure 6), and has been studied previously [116]. Previous works have demonstrated that the venous system exhibits mechanical changes in accordance with cardiac, respiratory, and autonomic physiological functions [119]. Muscular contraction and relaxation are the major functions contributing to the movement of blood from the veins back to the heart, as well as venous valves preventing back flow of blood. The difference in compliance between the arterial and venous systems, as well as the lower-pressure gradient, translates to the relative amplitude of the AC venous component of the PPG being smaller than the AC arterial component [125]. While this relationship is largely maintained over the body, PPG measurements at different body sites can result in stronger relative contributions from the venous system, such as the forearm versus the finger. The finger is arterially dominated while the forearm has a larger venous component [120]. Thus, the venous system can potentially add noise to a PPG due to vein pulsations and the variation of the amplitude of those pulsations across the body.

Shelley et al. demonstrates diastole variability in the PPG signal is related to peripheral venous pulsation [116]. The investigation conducted utilized a PPG sensor on the index finger alongside a radial catheter on the same hand. Measurements were taken on three patients under general anesthesia: a 72-year-old woman with osteoarthritis and nadolol-treated hypertension, a 40-year-old woman with a ruptured ectopic pregnancy, and a 57-year-old woman undergoing a suboccipital craniotomy with nifedipine-treated hypertension. Data were presented observing the variation in arterial waveform, central venous pressure, peripheral venous pulsation, and PPG signals. Peripheral venous pressure was monitored through an intravenous catheter with pressure. PPG signal was monitored both pre- and post-catheterization to verify that the catheter did not significantly affect the overall recorded signal. Continuous monitoring of both venous and artery contributions to the PPG waveform indicate strong correlation between the diastolic peak in the plethysmograph and peaks in the venous pulse at the peripheral location of the hand [116]. In two specific cases, a qualitative test was performed by applying light pressure to a site proximal to the measurement location on the upper arm and observing changes in three different stages of the venous pulse and PPG. Pressure in the venous system is observed to be significantly lower than the arterial system: below 20 mmHg has been reported [126]. Vascular compliance between the venous and arterial system can differ significantly, up to 10-fold [126]. Light pressure application upstream is used to occlude the low-pressure pulsation in the peripheral venous system. As the pressure is applied and released, the amplitude of the venous pulsation can be observed as being superimposed at the diastolic phase of the PPG curve with a slight time delay when there is no applied pressure, and no superposition when there is low pressure applied proximal to the hand. It appears that changes to diastolic amplitude due to the presence of the venous waveform can increase diastolic phase amplitude up to 40% of the total alternating amplitude of the PPG signal. In the case reports studied by Shelley et al., the significant relationship of the influence of the venous pulsation to the PPG waveform is observed at the peripheral location [116]. Although the specific changes to the waveform itself are not quantified, the observable effect of the venous system can alter conclusions made solely on the PPG signal without accounting for venous pulsation. The presence of the venous pulsation associated peak in the diastolic phase of the PPG waveform can present errors with identification and quantification of the dicrotic notch and diastolic features. Noninvasive measurement of venous pressure can help with diagnosing clinically relevant conditions such as congestive heart failure or valvular heart disease; however, separation of these signals is desirable [116].

Shelley et al. and Sami et al. both demonstrated the influence of the venous signal on SpO2, showing that forehead and earlobe pulse oximeters (respectively) produced a significantly biased variable component of the PPG determined to originate from central venous pressure (CVP) [125,127]. Sami et al. analyzed venous flow originating from a 72-year-old woman with ischemic cardiomyopathy and severe three-vessel disease. Shelley et al. studied data from forehead pulse oximeters originating from 25 patients (20 female, 5 male) undergoing elective gynecological and urological procedures that are otherwise healthy. The pulse oximetry values were both lower in cases where CVP contributed to the signal. In five of the twenty-five patients monitored by Shelley et al., prominent interference was observed by the venous component on forehead PPG signal when compared to signals observed at the finger [102]. Nijland et al. also confirmed these results in fetal lambs, where reflectance pulse oximetry SpO2 readings were significantly lower than fiberoptic SaO2 values. However, when the vessel was coagulated, this difference became negligible [128]. By having deoxygenated blood in the veins, a standard pulse oximeter will interpret there to be a higher ratio of deoxygenated blood to oxygenated blood, which yields a lower SpO2. All authors were able to eliminate the problem by applying pressure or otherwise occluding veins, a common practice in commercial oximeters.

Within a PPG waveform, venous pulsations can also be detrimental. They have been shown to artificially inflate the systolic peak amplitude and interfere with the rising systolic edge of the typical PPG waveform; an important parameter used in cardiovascular health determination [116]. The frequency components contributing to the perturbations in the signal are similar to the cardiac frequency. Venous pressure can also add low-frequency noise to the PPG waveform, the same effects seen by respiration, which is caused by venous anatomy [129,130,131]. Often between 0 and 0.5 Hz, this noise contributes to be baseline oscillations.

Eliminating venous contributions to PPG, as mentioned previously, is often done by application of contact pressure to the measurement site. However, it is possible that this will eliminate the arterial pulsation in hypotensive patients as well as increase pressure-induced injury risk to the measurement site [125]. This, along with the other effects of applied pressure, is explored in Section 4.3. Grabovskis et al. noted that the amount of pressure applied is often underreported and can manipulate the resultant PPG waveform [132]. This then affects clinical parameters similar to those reported by Takazawa [29]. They determine that the appropriate amount of pressure is such that the *b*/*a* ratio of the SDPPG is equal to 0.70. This means that the external pressure on the arterial wall is equal to the mean arterial pressure [132]. However, this is detrimental, since manipulating the waveform to a specific shape limits the useful data that can be extracted. Beyond contact pressure, the respiratory component has been eliminated via adaptive and non-adaptive filters allowing signal from approximately 0.5 to 4.0 Hz [133]. Additionally, previous works discuss variable-frequency complex demodulation, continuous wavelet transformation, and autoregressive modeling to decouple this signal from the arterial component of the PPG [22,120,127,132].

### 3.3. Body Site of Measurement

PPG systems have been developed for the fingers, wrist, brachia (upper arm), earlobe, ear cartilage, superior auricular region, esophageal region, and the forehead [68]. For fitness applications, the PPG system developed for the brachia is often used to monitor heart rate, but devices that can be placed on the wrist have increased in popularity due to them being commercial availability, their ease of use, cost, and portability. In clinical settings, the fingertip or ear lobe are more common locations due to the high vascularization found in these areas. While it is advantageous to have different devices for different locations, it is problematic when algorithms, processing techniques, and indices to assess cardiovascular risk are applied without considering the various anatomies at each of these locations. Anatomical variations in parameters such as skin thickness and blood basal perfusion will lead to changes in the AC and DC amplitude, and the duration of a PPG waveform. While a thick epidermis will attenuate light more than a thin epidermis will, the vascularization and perfusion of a given anatomy is particularly important in governing the magnitude of the AC component of the PPG. Thus, this section will discuss the impact of body site of measurement on the PPG waveform.

**Figure 7:**
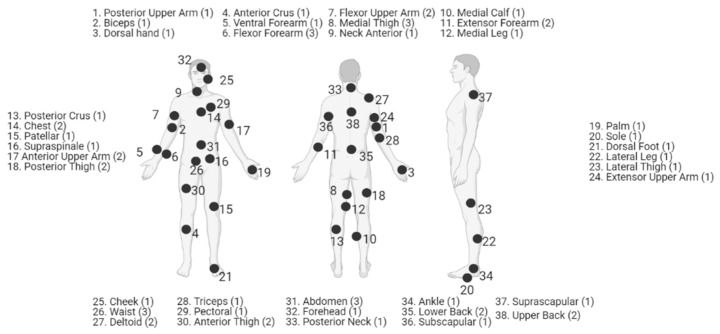
Relative total skin thicknesses across body sites for a sample of studies, from thinnest to thickest. Each anatomy also has (1), (2), or (3) next to its name, indicating the number of studies this body site was measured in (out of 5 considered works). Created with BioRender.com [80,81,140,142,143].

Changing skin thicknesses across the body lead to changes in the amount of light attenuated before it reaches microvasculature, such as the arterioles or an artery. This influences the signal amplitude and SNR of the resultant PPG waveform. The thickness of individual skin layers has been well characterized in a variety of ways; however, the skin as a function of anatomical location has been less well studied [134,135,136]. In general, skin found on tactile anatomies such as the finger will be thinner than skin found on anatomies such as the palm of the hand or the sole of the foot. In vivo high frequency ultrasound is the current standard for monitoring skin thicknesses, as old practices such as stained samples from biopsies or results postmortem often yield values with poor intersample agreement [137,138]. Overall, the literature has shown that the fingertips have the thinnest skin, followed by the forearm, dorsal hand, cheek, and forehead [139]. The skin thickness for 38 anatomical locations has been ranked based off 5 separate works (Figure 7). However, there exists tremendous variation even within a given body site caused by factors such as age, gender, BMI, sun-damage, and experimental methodology, leading to coefficients of variation up to 40% [139,140,141]. Skin thickness is not the only body site-dependent factor causing variations in PPG results. In order to quantify the effect of body site on PPG, one must also analyze changes in blood supply and basal perfusion. The literature has analyzed their cumulative effect on PPG.

A common difference across anatomies is basal perfusion, which directly affects PPG signal amplitude. Tur et al. utilized laser doppler velocimetry (LDV) and PPG to assess variations in blood perfusion across the body for 10 healthy men between 20 and 30 years old. Both tools in combination provided assessment of both blood velocity and blood volume. It was found that the hand and the face had significantly higher (*p* < 0.01) perfusion values than the trunk, upper, and lower limb. Additionally, the side of the trunk was found to have very low perfusion. Finally, sites within the face and hand did not yield widespread statistical difference between themselves. The back of the ear and earlobe yielded significantly higher PPG signal amplitude than the hand and postauricular region (i.e., neck behind the ear), but LDV values and comparisons to the fingertips and the rest of the face were not significant. These data support the hypothesis that the ear and the fingertips will yield the largest PPG signal amplitude [144]. This study did not look at the wrist, but as mentioned previously, authors found the forearm to have lower perfusion levels as compared to the face and hand. While these results provide information about superficial vasculature 1–2 mm deep in the skin, it did not provide information about accompanying variations in skin thickness across sites. Overall, it has been found that locations on the head and finger provide the greatest PPG amplitude [95,145]. Locations around the ear are less uniformly reported in the literature; however, this likely is caused by inconsistencies across works, as some state “ear” and others provide more detail. Fallet et al. found via iPPG that for all wavelengths of light, the forehead is superior to the cheeks and the whole face for determining heart rate. The forehead yielded the greatest power at a frequency matching a reference ECG, likely due to the increased perfusion to the area [146].

Beyond PPG amplitude, the aforementioned anatomical variations lead to changes in PPG waveform. First, it is important to note bilateral symmetry. Allen and Murray have noted that PPG waveforms are largely similar between bilateral anatomies at the ear, finger, and toe at rest, according to their study featuring 116 (68 male, 48 female) healthy Caucasian subjects with a median age of 43 [90]. Additionally, PTT is also similar bilaterally at the ears and finger, while a small difference exists at the toes [91]. This is important as studies often do not note the bilateral location of the sensor (if relevant) or place the sensor on the dominant hand [147,148]. These studies demonstrate that a given left and right extremity will give the same signal, save for differences such as motion artifacts.

Rajala et al. analyzed the waveform and pulse arrival time of PPGs taken at the wrist and the finger of 30 subjects (19 males, 11 females, average age of 42) [26]. It was found that the wrist significantly (*p* < 0.01) had a greater full-width half-max than the finger. The authors ascribed this difference to the flat shape of the wrist PPG, whereas the finger PPG more closely resembles a traditional PPG with a noticeable dicrotic notch. Additionally, the authors noted a consistent increase in signal amplitude in the wrist PPG compared to the finger PPG. They hypothesized that this effect could be due to an increase in wrist temperature due to the fabric component of the PPG sensor. Hartmann et al. performed a similar study in 36 healthy subjects (12 male, 24 female, mean age of 33), looking at the peak point position, dicrotic notch time, and reflection index at the forehead, earlobe, arm, wrist (upper, under), and the finger [95]. The finger yielded a significant peak point position, meaning a shorter systolic rising edge (*p* < 0.001 for all sites except under the wrist, which had *p* = 0.04). Dicrotic notch duration at the finger was not significantly different from measurements under the wrist and forehead, but was different from the earlobe, arm, and wrist. Finally, the finger was shown to have a significantly lower (*p* < 0.001) reflection index.

These differences across anatomies are crucial to note when using PPGs to derive clinical diagnoses/prognoses. For example, Alty et al. demonstrated the use of crest time to classify CVD, where the crest time is directly related to the systolic rising edge which is significantly shorter in the fingertip [149]. Takazawa et al. named numerous ratio metric parameters as a function of the APG, the second derivative of the PPG. Many of these parameters involve components of the reflection index, which the above study showed can be different at various anatomies [29]. Thus, not only should researchers be consistent to the same anatomy across studies, but it is possible that different anatomies are better at providing diagnostic/prognostic-friendly waveforms for various applications. Nillsson et al. demonstrated that this is the case for respiratory rate. It was found that the forearm had a high respiratory rate spectral power content compared to anatomies such as the finger, which had signal primarily derived from heart rate [130].

Overall, the literature has demonstrated that different anatomies demonstrate PPGs with varying waveforms. Additionally, the thickness of skin and blood perfusion will play a role in the strength of the signal able to be obtained. Due to the presence of a distinct dicrotic notch and low skin thickness, the finger is likely to be a successful anatomy that can be used with consistency. It remains to be seen if diagnostic predictor parameters such as those presented by Takazawa retain their statistical and predictive power across anatomies and local variables such as body temperature [29].

### 3.4. Local Body Temperature

Thermoregulation is an important component of homeostatic function. Large shifts in temperature typically occur in response to external stimuli, such as exercise and contact. Subtle temperature changes can follow regular patterns associated with normal physiological functions. For example, Allen et al. studied three adjacent fingers of the hand of 15 healthy males measuring PPG, laser doppler flow (LDF), and skin temperature changes following a deep inspiratory gasp of air. They reported a 2.6-times increase in PPG amplitude, 93% decrease in LDF flux, and median decrease of 0.089 degrees Celsius [150]. The body’s thermoregulatory response to stimuli includes vasoconstriction/vasodilation, which could lead to a delayed response of temperature in the skin. Thus, while the pulsating components of PPG are related to arterial blood volume, the non-pulsating component is a function of the average blood volume, respiration, the sympathetic nervous system, and thermoregulation [151]. As such, typically filtered out PPG components can provide information surrounding thermoregulatory blood flow, one example being through arterio-venous anastomosis shunt vessels [151]. Furthermore, while studying the effectiveness of PPG on identifying limb ischemia among men and women of an average age of 70 years old, Carter and Tate reported that PPG amplitude is significantly correlated (r = 0.550; *p* < 0.001) to skin temperature of the toe, as body cooling leads to reductions in PPG wave amplitude [152]. Lindberg et al. found that PPG amplitude showed a direct response to skin temperature elevation, especially in the finger skin of 10 Caucasian young adults (aged 22–30) using three different arrangements of PPG probes with different source/detector separations [153]. These correlations of PPG with body temperature should be noted among varying demographic groups. For example, Iacopi et al. found the skin of the foot of obese patients to be about 7 degrees Celsius higher than the foot of nonobese patients, and thus caution should be noted if PPG is measured at the foot [60].

Khan et al. investigated the effects of finger temperature on PPG signal on 20 healthy adult volunteers (24.5 ± 4.1 years of age), reporting a reduction in PPG Root Mean Square values as ambient temperatures went from warm to cold temperatures [154]. The authors conclude that PPG quality is improved with warm temperatures, yet do not mention the effect of thermoregulatory responses [155]. Hahn et al. studied the effect of cold exposure on arterial PPG among 10 heathy volunteers and 10 individuals with systemic sclerosis. They report significant reductions in PPG pulse wave amplitudes (*p* < 0.0001) between the groups both at rest and after cooling the finger to 16 degrees Celsius [156]. Askaraian et al. reported that while measuring PPG with a submerged finger of 20 healthy volunteers aged 18 to 80 years old, they found that a drop in 40 degrees Celsius contributed to a 12 dB drop in PPG signal amplitude [157].

Temperature drops also seem to result in decreased accuracy of heart rate estimation. Jeong et al. found that local skin surface temperature changes affect PPG components of 16 healthy subjects aged 23–30 years (26 ± 2.1) and BMI ranging between 20 and 26 (23 ± 4.8), hence recommending that temperature be monitored in order to reliably evaluate cardiovascular parameters [158]. Zhang et al. demonstrated how PPG reproducibility is affected by cold exposure and acclimatization after middle finger submersion in 9 ± 2 °C water for 2 min and comparison with index finger as a reference [159]. Significant changes in DC and AC amplitudes of the PPG pulse indicate that the mild cold exposure has a substantial effect on finger blood circulation. The authors suggested that mild cold exposure may have a delayed effect on PTT due to cold-induced vasodilatation and could be a potential source of error [159]. Thus, while physiological temperature changes due to thermoregulation may impact a signal, studies detailing changes caused by external temperatures also illustrate significant PPG impact.

Aside from temperature changes due to the subjects’ physiological state, abrupt changes in ambient temperature could affect the PPG system components, and thus the PPG signal. There are also PPG instrumentation aspects that have strong temperature dependence. For example, many of the photodetectors are subject to creating artifacts from sensor-tissue motion and sensor deformation changes [160]. For silicon photodiodes, the absorption coefficient increases with temperature, and thus the detectors will absorb less light at higher temperatures, inducing an apparent shift in PPG amplitude [161]. Higher temperatures also generate higher thermal noise in the detector. Drift current varies directly with temperature when photodiodes are used in photovoltaic mode [161].

It has been shown that the temperature of the body site where PPG data are being collected can influence the resultant waveform amplitude, and even the time between waveforms. This is summarized in Table 5. However, many of these studies suggested that external temperature has a much more significant effect on the PPG waveform. Thus, beyond variation found within individuals and changing physiologies, external factors can also significantly impact RCIM PPG.

## 4. External Factors

The previous two sections discussed sources of error pertaining to the subjects of cardiovascular monitoring. This next section explains sources of error that originate from the environment and factors outside of the patient’s characteristics. These are motion artifacts, ambient light, and applied pressure to the measurement site. While the previous sections are relevant to discerning and evaluating the PPG waveform, this section more heavily discusses obtaining an accurate signal.

### 4.1. Motion Artifacts

PPG sensors are used in settings where a person is sedentary or in motion. Within all settings, motion artifacts are picked up by the sensor and cause fluctuations within the collected signal. During sedentary moments, respiration rate, thermoregulation, and sympathetic nervous system activity cause the DC baseline to wander [162]. Adjusting positions or tapping a finger can be considered a micro-motion, while a macro-motion is performing an exercise such as walking or jogging—both types of motion cause more significant fluctuations to the signal than sedentary motions. With macro-motions, there are different grades of intensity, and each affects accurate signal acquisition. All these motions range between 0.1 and 20 Hz, which is within the frequency range for heart rate (1–4 Hz) [163]. With the addition of motion artifacts within a PPG signal, results for SpO2, heart rate, and other PPG dependent measurements can be skewed. This can create false alarms or inaccurate readings. Another issue is when motion is cyclical or periodic. Heart rate tracking devices will pick up on the cyclical motion and mistake it for the cardiac cycle, causing false readings as well [46]. With the increase in commercial heart rate monitoring devices, there is an increased need for motion artifact identification and elimination.

Motion artifacts can affect the acquired signal differently based on the location of the sensor along with what wavelength of light is used. Maeda et al. measured both IR and green wavelengths along the upper arm, forearm, wrist, and finger [162]. They found the upper arm had the lowest artifact ratio, which is the ratio of the magnitude of the PPG signal after a motion and the magnitude of the PPG signal before the motion. The green sensor also had a lower artifact ratio for all locations compared to the IR sensor. The highest correlation coefficient between the PPG signal and a chest ECG was that of the green sensor on the upper arm. Lee et al. saw similar results regarding the wavelength used [164]. They looked at the correlation coefficient and ΔSNR for blue (470 nm), green (530 nm), and red (625 nm). With the longer wavelength, red had a significantly bigger ΔSNR than green or blue. It was determined that green was the best option due to its higher correlation coefficient, but blue is an alternative option due to its significantly similar ΔSNR as green. With their longer wavelengths, red and IR have deeper penetration depths compared to blue and green, making them prone to more motion artifacts such as the factors that directly affect the DC baseline [162,164]. Aside from location and wavelength, the intensity of motion also plays a role in how accurate heart rate monitoring is.

In order to understand how motion artifacts affect the accuracy of heart rate monitoring, researchers have tested off-the-shelf health and fitness trackers and compared them to a chest ECG during different intervals of motion. All the discussed trackers are designed to be worn on the wrist. Jo et al. compared the accuracy of the Basis Peak (BP) and Fitbit Charge HR (FB) during high-intensity events where the mean ECG HR was >116 bpm, and low-intensity events where the mean ECG HR was <117 bpm [165]. Overall, they discovered that the BP performed better, in relationship to the ECG, (r = 0.92, *p* < 0.0001) than the FB (r = 0.83, *p* < 0.0001). During the low-intensity events, the BP provided accurate heart rate readings (r = 0.84, *p* < 0.05), while the FB had a moderate correlation (r = 0.73, *p* < 0.05). High-intensity events caused the BP to have a weaker correlation (r = 0.77, *p* < 0.05), whereas the FB had the weakest correlation (r = 0.58, *p* < 0.05). These results align with an assessment done by Stahl et al., where they measured the mean absolute percentage error values (MAPE) of BP and the FB to be 3.6% and 6.2%, respectively [166]. Stahl et al. also looked at the Scosche Rhythm, Mio Alpha (MA), Microsoft Band, and TomTom Runner Cardio (TT). When going from a resting phase to a low-intensity walking phase, they found that the correlation coefficient went down but had slowly rebounded when moved into a more intense walking phase. They also reported that during the high intensity running stage, they saw some of the lowest MAPE from the MA (0.82%), the TT (0.97%), and the BP (3.28%). This conclusion showed that during intermediate and low intensity motions, the accuracy is dependent on the device. In another study, performed by Dooley et al., the comparison was made between the Apple Watch (AP), FB, and Garmin Forerunner 225 (GF), where the AP was considered the best in terms of MAPE (1.14–6.70%), while the FB and the GF had a MAPE of 2.38–16.99% and 7.87–24.38%, respectively [167]. The AP (*p* = 0.84) and GF (*p* = 0.35) had no significant difference in HR readings from the ECG during vigorous intensity. All had a significant difference during low-intensity events. This shows that, depending on the exercise, each tracker will have a different accuracy, which could be attributed to the different noise-canceling algorithms used on board. Bent et al. confirmed that the wearable device and activity condition are significantly correlated in their study [46]. There was a significant difference between research-grade and consumer-grade wearables. The mean absolute error (MAE) for all motion phases was better for the consumer wearables than the research wearables. This study also showed that the Apple Watch 4 had the highest accuracy with an MAE of 2.7 BPM at rest and 4.6 BPM during the walking phase, confirming the results from Dooley et al. All the studies have shown that motion artifacts cause varying accuracies depending on motion intensity and the type of heart rate monitor used. Being able to detect the motion artifact regardless of motion intensity is crucial for more accurate heart rate measurements.

There are several ways of detecting noise or motion artifacts that do not rely on secondary sensors, which include using filters with cross-correlation, analyzing the morphology of the signal, and higher-order statistics in both the frequency and time domain. Karlen et al. created an algorithm with 96.21% sensitivity and 99.2% positive predictive value for good pulses using repeated Gaussian filters and cross-correlation [168]. The signal is analyzed in the time and frequency domains and then cross-correlated to determine whether the pulse has a high or low signal quality index (SQI). The correlation utilizes the rising slopes of at least three previous pulses along with applying repeated Gaussian filters to predict where the next pulse should be. If the pulse does not fall within the range of prediction, the pulse is deemed as bad (low SQI) and could be eliminated from HR calculations. With this algorithm, Karlen et al. were able to distinguish where motion artifacts occurred in a beat-to-beat manner. Another form of predicting motion artifacts is to dissect the signal using morphology. Sukor et al. focused on locating the pulse amplitude, the differences in trough depth between successive pulses, and the pulse width [169]. They found their algorithm to have an accuracy of 83 ± 11%, a sensitivity of 89 ± 10 %, and a specificity of 77 ± 19%. When comparing their algorithm to an ECG, they were able to lower the error in heart rate readings to 0.49 ± 0.66 bpm, whereas without the algorithm, the error was 7.23 ± 5.78 bpm. Chong et al. also worked on identifying four time-domain characteristics while using a support vector machine to build a decision threshold to classify clean and corrupt PPG signals [170]. They were able to achieve an accuracy of 93.7% during a daily motion experiment, and they saw a significant reduction in error for SpO2 and heart rate measurements. Karlen et al., on the other hand, identified time-domain characteristics by using pulse segmentation to determine the up-slopes of the PPG signal [171]. Their application was created for mobile PPG measurements instead of wearable. They were able to achieve a positive predictive value of 96.68% and a sensitivity of 98.93%. Like the others, Coucerio et al. used time-domain analysis, but they also implemented period domain analysis to identify 26 features across both domains [172]. They were able to achieve a specificity of 92.7% and a sensitivity of 82.7%. The last detection method is to use higher-order statistics done by Krishnan et al. and Selvaraj et al. [173,174]. Kurtosis and Shannon Entropy were the measurements used by Selvaraj et al. to determine motion artifacts, and they were able to perform an accuracy of 88.8% in a laboratory setting [174]. Krishnan et al. used kurtosis and skew in the time domain while also analyzing frequency domain kurtosis and quadratic phase coupling [173]. They discovered skew and kurtosis measurements in the time domain to be higher when there was corruption, and the frequency-domain kurtosis was smaller when there was corruption. The quadratic phase was present in corrupted signals. All the parameters could then be used to eliminate sections of the PPG signal that were corrupted with motion artifacts. The discussed detection methods are all real-time ways to detect and eliminate sections of acquired PPG signals that contain corrupted areas due to motion artifacts. This will help to keep heart rate and SpO2 measurement error lower.

As implemented by consumer-grade HR monitors, secondary sensors can also be integrated into PPG sensors to detect motion. These include accelerometers [175,176,177,178,179], gyroscopes [179,180], piezoelectric transducers [181], or the utilization of another wavelength of light as a motion reference [182,183]. Each of these sensors is used to detect and then reduce or eliminate noise so a clean signal can be reconstructed. Accelerometers and gyroscopes are the standards, but when trying to measure micro motions such as finger tapping, accelerometers and gyroscopes do not always pick up on the motion because the wrist remains still. Both the piezoelectric transducer and another wavelength of light can pick up these finer motions [181,182]. The algorithms used to detect motion with a secondary sensor also use adaptive filters which have been used independently of sensors as noise reduction techniques but are better suited to work in conjunction with secondary sensors. Table 6 lists adaptive filters used to reduce motion artifacts. 

With the use of adaptive filters and secondary sensors, motion artifacts can be identified and eliminated, or they can be identified and reconstructed to restore the original PPG signal, void of all motion artifacts. This has been shown to decrease the error in heart rate and SpO2 measurements.

### 4.2. Ambient Light

PPG signals are low-amplitude signals and have a normal pulse frequency within the range of 0.5 to 4 Hz [195,196]. The interference of ambient light with the PPG signals can lead to an inaccurate heart rate estimation. The ambient light intensity can be a zero frequency (e.g., DC), such as sunlight, or at a variable frequency, as from room lights (e.g., 60 Hz in the US) and is generally multiple orders of magnitude larger than the pulsatile (AC) component of the PPG, which can lead to signal saturation. Thus, ambient light rejection is important to preserve the efficacy of PPG sensors [197,198]. In 1991, it was found that commercial pulse oximeters had measurement error caused by ambient light [199]. Since then, development for RCIM has trended towards PPG devices that operate with applied pressure on the body and/or in body sites that help block other light, ensuring that the detectors are receiving maximum transmitted/reflected light from only the device.

An approach to reduce the effect of ambient light in PPG measurements can be seen in the work of Wang et al., which proposed an ear-worn sensor operating in reflective mode with multiple light sources and detectors. The optical components in this sensor include light sources which are DLED-660/905, DLED-660/940 and PDI-E835. It is important to note that the photodetectors, BPW34 and BPW34FS from Siemens (Munich, Germany), used in this sensor come with day-light filters. In addition to that, the components of the sensors were optically shielded by embedding them into the base of the sensor and separating them with an opaque medium [200]. This method was found to be effective in shielding from noise due to ambient light, as the DC level of the PPG signal was considerably low (measured to be less than 2 nA) when the LEDs were switched off. Patterson et al. proposed a flexible PPG sensor with a design that can minimize the effects of ambient light and other electromagnetic interference. This system with an API PDI-E832 light source, which is a dual LED emitting at 660 and 905 nm, and an API PDV-C173SM photodetector, measures the PPG signal from auricular region. The opto-electronic modulation scheme in this flexible PPG sensor helps to eliminate ambient light noise through the multiplexing technique and sampling of light level by keeping the LEDs off for a fixed time period and later subtracting it out from the desired signals. Apart from its electronic system design, the arrangement of the source and detector in this sensor also contributes to eliminate the interference of ambient noise to the signal. This includes encasement of the active area of the photodetector in a red plastic to filter out ambient light and addition of 2 mm wide foam between the source and detector to prevent direct coupling of light from the LED to the detector [201]. Selective filtering of unwanted signals (above and below 0.5 to 4 Hz frequency range) is another method followed to eliminate the noise due to ambient light. With a low bandwidth of around 5 Hz, PPG signals can be extracted out from high frequency noise using a low-pass filter and from low frequency noise using a high-pass filter (e.g., to reject 60 Hz room lights and to reject DC sunlight). Various attempts are also made in the PPG sensing circuitry level to effectively reject DC photocurrent. A transimpedance amplifier (TIA) associated with PPG sensors converts and amplifies the weak photocurrent from the PD to a differential voltage. An effective DC photocurrent rejection circuit proposed by Wong et al. by developing an integrated TIA with bandpass response in a NIR wearable sensor is an example. The dual loop TIA in this design acts as a high pass filter by achieving a reduction in its cutoff frequency as low as 0.5 HZ, while the other loop adaptively adjusts the DC photo current and prevents sensor saturation [202].

The accuracy of PPG measurements could be limited due to the interference of environment noise such as ambient light. This can be effectively compensated by various methods like optical shielding of the transducer, selective filtering of noise outside the PPG bandwidth, and with correlated double sampling. Modifications in the signal processing method at the signal amplifier can also successfully reject DC photocurrent in PPG sensors.

### 4.3. Applied Pressure to Measurement Site

Discrepancies in PPG signals can arise where variations in applied pressure influence the resulting waveform. This can result in an increase or decrease in amplitude along with a shift in offset. With a low applied external pressure from the sensor, the waveform exhibits lower SNR with a low AC amplitude primarily because of a longer optical path length through the tissue and a lower reflectance of photons due to high tissue absorption [203]. With increased applied pressure, the AC amplitude begins to increase due to a decrease in optical path length through the tissue and the approach of the transmural pressure to zero. The transmural pressure is defined as the pressure difference between the mean intra-arterial pressure on the vessel (e.g., artery or arteriole) wall and the contact pressure. When the transmural pressure reaches zero, the AC amplitude reaches its maximum [204,205]. As the external pressure exceeds the point where the transmural pressure is no longer zero, the vessel begins to occlude, and the AC amplitude begins to decrease until there is no longer a signal where the vessel is occluded. Different vasculature will occlude at different amounts of pressure, also contributing to perturbations in the signal. With the change in transmural pressure, there is also a related compliance change in the affected vessel. The vessel reaches maximum compliance when the transmural pressure is zero [205].

Several characteristics of the PPG waveform are affected by changes in contact force, including; the AC amplitude, the DC offset amplitude, the ratio of AC/DC amplitudes, and the normalized pulse area. As reported by Teng and Zhang, with an applied force at the finger of 0.2–1.8 N, there was an increase in AC amplitude and AC/DC ratio until the parameters reached a peak and then decreased, while the pulse area decreased, and the DC offset increased [206]. The change in these fundamental features can affect other measurements such as the *b*/*a* ratio, derived from the a and b peaks of the second derivative of a PPG signal, used to characterize arterial stiffness [132], the relationship between the frequency response of a PPG signal and a blood pressure signal [207], or the pulse transit time (PTT) which is used as an indicator for fluctuating stiffness or elasticity in arteries and blood pressure [208]. Grabovskis et al. reported with an applied pressure range of 0–15 kPa, there was a variation of over 300 percent in the *b*/*a* ratio [209]. When an optimal pressure was reached, the variation coefficient dropped to less than 5 percent. Using the frequency response of the PPG signal taken at the finger, Hsiu et al. worked to understand how the relationship between the first five harmonics of the PPG signal and corresponding blood pressure signal varied with an applied pressure of 0–200 mmHg [207]. At 60 mmHg, there were the greatest R^2^ values for four out of five harmonics, and with the different applied pressures, the R^2^ values ranged from 0.13 to 0.77 for the first harmonic. Regarding PTT, Teng and Zhang discovered that, with a contact force of 0.1–0.8 N applied at the finger, the PTT measured from the R peak of an ECG and the peak of the second derivative of the PPG increased significantly (*p* = 0.014) until the estimated transmural pressure approached −0.1 N, after which the PTT remained roughly constant [208]. For the PTT measured from 50% of the pulse amplitude, there was a significant increase in PTT (*p* = 0.038) before reaching a constant at −0.1 N transmural pressure. Lastly, for the PTT calculated at 90% of the pulse amplitude, there was a similar increasing trend for the PTT but without a significant difference (*p* = 0.107). The PTT leveled out to a constant value for a transmural pressure of around 0.1 N. Within all the studies, infrared light-emitting sources were used to study the effects of contact pressure. Spigulis et al. worked to understand at what pressure the signal amplitude would be at maximum and when the vasculature would occlude at wavelengths of 405, 532, 645, 807, and 1064 nm. Since longer wavelengths going from the visible to NIR penetrate deeper, it took larger pressures before occlusion occurred, since the deeper arteries with higher mean arSPOterial pressure (MAP) were the ones probed with the longer wavelengths [210]. For shorter wavelengths, the occlusion pressure was lower, as more superficial arterioles with low MAP were in the light path at these wavelengths. Overall, the above studies show that inaccurate PPG signal acquisition due to varying contact pressures can lead to inaccurate secondary measurements. Therefore, integrating a solution to produce constant contact pressure or being able to measure the pressure in PPG devices will produce more consistent and repeatable measurements.

Integrating a sensor that can measure the applied force could help to standardize PPG measurements such that the applied pressure does not skew the signal. For example, Nogami et al. designed a PPG sensor with an optical displacement sensor comprised of a vertical-cavity surface-emitting laser, a mirror, and a photodiode [211]. Specifically, there is a compressible frame with the mirror mounted to it and as a force is applied, the intensity of light reflected from the mirror on to a photodiode changes in accordance with the applied pressure. Another solution is to integrate a flexible thin-film force transducer (FlexiForc A201, Tekscan Inc., Boston, MA, USA) between the PPG sensor and the designed fastener, as done by Grabovskis et al. and Sim et al. [209,212]. Grabovskis et al. reported a less than 3% coefficient of variation within a subject at the single measurement site when determining the optimal pressure needed to determine an unloaded artery. Sim et al. utilized the force transducer as a feedback mechanism within their system. A force regulator consisting of a thermo-pneumatic actuator would heat a layer of expandable fluid that pushed down upon the force transducer and PPG sensor. Without force regulation, the coefficient of variation between five posture stages was calculated at 50.9%. The addition of the force regulator dropped the coefficient of variation to 1.8%. Rhee et al. created a finger-ring system that utilized a polyester braided elastic band to mount their sensor [213]. This band would apply a skin pressure of about 75 mmHg and, due to its compliance, it would hold steady the applied pressure while the finger was in different positions. Another force measuring modality to integrate with a PPG sensor is a Force Sensing Resistor (FSR, Interlink Electronics, Camarillo, CA, USA), as done by Santos et al. [214]. Liu et al. implemented a fiber Bragg gating to measure applied pressure [215]. The Bragg wavelength will measurably shift in relationship with the applied pressure to the sensor. Utilizing this modality, they demonstrated that, when pressures are kept within the range of 5–15 kPa, there is less than 2% error in the SpO2 measurements.

## 5. Summary

This review focused on the impediments to remote and continuous PPG devices. PPG is a common tool used to monitor cardiovascular health in controlled environments. However, only a limited number of RCIM PPG devices have FDA approval. A review of the literature shows that the difficulty in creating such a device partially originates from the many sources of noise that researchers encounter. Here, we compiled and evaluated sources of noise found in published works and utilize understandings of photoplethysmography and light/tissue interactions to summarize findings in order to provide guidance for future PPG-based devices. As shown in Table 7, we found that sources of noise can be divided primarily into three categories: individual variation, physiological processes, and external perturbation. While many sources of noise had documented potential solutions, few had a comprehensive solution and even our understanding of how conditions such as obesity affect the skin and cardiovascular monitoring are still developing. Future research towards a RCIM PPG device for true regulated health monitoring should incorporate larger studies that are inclusive of the noise sources across diverse populations discussed herein.

## Figures and Tables

**Table 1:** PPG-based remote and continuous/intermittent monitoring (RCIM) devices with Food and Drug Administration (FDA) status and popular fitness devices.

Device	Company	FDA Status	PPG-Derived Parameters	Release Year	Notes
**Devices with FDA Status**					
Samsung Gear S2/LIVMOR Halo™	Samsung/LIVMOR	Cleared	Heart rate variability	2020	Samsung Gear S2 is FDA approved to assess heart variability only with the LIVMOR Halo™ Detection System
BB-613	BioBeat	Cleared	Oxygen saturation of arterial hemoglobin (SpO2), pulse rate, and changes in blood pressure	2019	Available as a wristwatch or patch
Loop	Spry Health	Cleared	Heart rate, respiration, pulse oximetry	2019	-Wristwatch designed for individuals with chronic obstructive pulmonary disease
Wearable	Current Health	Cleared	Pulse rate, SpO2	2019	-Upper arm patch -Part of the Current Health Full-Service Remote Healthcare Platform
EQO2 Lifemonitor	Equivital	Cleared	SpO2	2013	-Worn as an insert in a chest harness -Works with ECG and other measurements
Everion	Biofourmis	Exempt	SpO2, Heart Rate	2017	-Biofourmis is currently performing clinical validation for Heart Rate Variability and Respiration Rate
**Popular Fitness Devices**					
Fēnix 6 Series	Garmin	None	SpO2, Heart rate	2019	-Heart rate variability available with chest heart rate monitor
Xiaomi Mi Band 5	Xiaomi	None	Heart rate, Heart rate variability	2020	-Also measures “respiration information”
Suunto 9	Suunto	None	Heart rate, Heart rate variability	2018	-Heart rate variability for sleep quality
Apple Watch Series 6	Apple	Cleared (ECG only)	SpO2, Heart rate, Heart rate variability	2020	ECG clearance is for irregular rhythm notification
Versa 3	Fitbit	Pending (ECG only)	SpO2, Heart rate, Heart rate variability	2020	Heart rate measurement is for rest conditions only

**Table 2:** Obesity-induced effects on PPG.

Parameter	BMI-Induced Changes	Presumed Effect on PPG	Relevant Work	Reference
Skin thickness	Skin thickness increases as BMI increases	Decreased signal resolution and intensity	Iacopa et al., 2020 Altintas et al., 2016 Boonya-Ananta et al., 2021	[36,60,72,77]
Blood flow	Baseline cutaneous blood flow increases in obese children, dermal blood cell flow increases in overweight individuals, cutaneous blood flow increases in the nailfold of obese children	Decreased signal resolution and intensity	Chin et al., 1999 Czernichow et al., 2010 Altintas et al., 2016	[61,63,72]
Capillary Density	Capillary density decreases as BMI increases	Increased signal resolution and intensity	Francischetti et al., 2011	[62]
Oxygen saturation	Oxygen saturation decreases as BMI increases	n/a	Petrofsky et al., 2015	[67]
Trans-epidermal water loss	TEWL increases as BMI increases	Increase PPG intensity and resolution (NIR and IR only)	Rodrigues et al., 2017	[69]

**Table 4:** Gender-induced changes on PPG.

Physiological Characteristic	Gender Discrepancies	Effect on PPG Signal	Relevant Work	Reference
Average Heart Rate	Higher average heart rate in females	Higher average heart rate yields higher average frequency content of the signal	Proctor et al., 1998	[99]
Heart Mass	Greater heart mass in males	Increased heart mass yields lower heart rate which yields lower frequency content of the signal	Prabhavathi et al., 2014	[100]
Blood Pressure	Higher mean blood pressure in males	Increased blood pressure increases PWV	Reckelhoff et al., 2001 Nye et al., 1964	[101,113]
Radial Artery Diameter	Larger average radial artery in males	Increased diameter increases flow rate which is an increase in PWV	Joannides et al., 2002	[102]
Arterial Stiffness	Greater arterial stiffness in pre-puberty and post-menopausal females	Increased arterial stiffness increases PWV and increases *b/a* ratio	Joannides et al., 2002 Ahimastos et al., 2003 Von Wowern et al., 2015	[102,104,114]

**Table 5:** Factors that affect local body temperature that affect PPG.

Temperature Increase/Decrease	Effect on PPG Signal	Relevant Work	Reference
Increase	Increase PPG amplitude and total signal	Allen et al., 2002	[150,153,154,155]
Decrease	Decrease in PPG waveform amplitude, decrease PTT	Lindberg et al., 1991 Hahn et al., 1999 Askarian et al., 2019 Zhang et al., 2006	[152,156,157,159]

**Table 6:** Adaptive filters utilized to eliminate or reduce motion artifacts independently and in conjunction with secondary sensors. This is not an all-inclusive list.

Filter	Relevant Work	Reference
Least Mean Square	Chan et al., 2002	[184]
Recursive Least Squares	Gibbs et al., 2005 Khan et al., 2015 Lee et al., 2010	[175,176,185]
Normalized Least Mean Squares	Han et al., 2007 Casson et al., 2016 Lee et al., 2010 Yousefi, 2013	[177,179,185,186]
Kalman Smoother	Lee et al., 2010	[185]
Spectrum Subtraction	Zhang et al., 2015 Zhang et al., 2015	[178,187]
Continuous Wavelet Transform	Zhang et al., 2019 Bousefsaf et al., 2013 Teng et al., 2003	[182,188,189]
Independent Component Analysis	Lee et al., 2020 Krishnan et al., 2010 Kim et al., 2006 Holton et al., 2013	[183,190,191,192]
Principal Component Analysis	Holton et al., 2013 Motin et al., 2017	[192,193]
Singular Value Decomposition	Lee et al., 2020 Reddy et al., 2007	[183,194]
Empirical Mode Decomposition	Yousef et al., 2012 Zhang et al., 2015 Motin et al., 2017	[88,187,193]

**Table 7:** Summarized noise sources, effect, and solution.

	Section	Sources of Noise	Impact	Mitigation Technique
Individual Variation	Skin Tone	Melanin absorption	Decreased signal intensity	PPG Wavelength Selection
	Obesity	Blood flow, skin thickness, capillary recruitment, trans epidermal water loss, oxygen saturation	Decreased signal intensity, modified PPG waveform	None found in literature
	Age	Skin thickness, vessel compliance, capillary recruitment	Change in signal intensity, modified PPG waveform	Calibration
	Gender	Cardiovascular baseline differences, skin thickness, vessel size	Change in signal intensity	Calibration
Physiology	Respiratory Rate	Low frequency noise	Modified PPG waveform	High pass filter
	Venous Pulsations	Reduction in overall signal, low frequency noise	Modified PPG waveform	High pass filter, apply pressure
	Local Body Temperature	Cold temperatures diminish PPG amplitude	Change in signal intensity	Calibration
	Body Site	Signal amplitude and PPG waveform shape	Change in signal intensity, modified PPG waveform	Calibration
External Factors	Motion Artifacts	High and low frequency noise	Change in signal SNR	Filters and secondary sensors
	Ambient Light	Increased Noise	Change in signal SNR	Optical shielding and selective filtering
	Applied Pressure	Reduction in PPG amplitude and SNR	Change in signal SNR, modified PPG waveform	Apply optimal pressure for high SNR, without affecting waveform features
